# Incidence of and risk factors for perineal trauma: a prospective observational study

**DOI:** 10.1186/1471-2393-13-59

**Published:** 2013-03-07

**Authors:** Lesley A Smith, Natalia Price, Vanessa Simonite, Ethel E Burns

**Affiliations:** 1 Department Social Work and Public Health, Faculty of Health and Life Sciences, Oxford Brookes University, Jack Straws Lane, Marston, Oxford, OX3 0FL, UK; 2 Department of Obstetrics & Gynaecology, Women’s Centre, Oxford University Hospitals Trust, Oxford, OX3 9DU, UK; 3 Department of Mechanical Engineering and Mathematical Sciences, Faculty of Technology, Design and Environment, Oxford Brookes University, Wheatley Campus, Wheatley, Oxford, OX33 1HX, UK

**Keywords:** Vaginal delivery, Perineal trauma, OASIS, Prospective study

## Abstract

**Background:**

Our aim was to describe the range of perineal trauma in women with a singleton vaginal birth and estimate the effect of maternal and obstetric characteristics on the incidence of perineal tears.

**Methods:**

We conducted a prospective observational study on all women with a planned singleton vaginal delivery between May and September 2006 in one obstetric unit, three freestanding midwifery-led units and home settings in South East England. Data on maternal and obstetric characteristics were collected prospectively and analysed using univariable and multivariable logistic regression. The outcome measures were incidence of perineal trauma, type of perineal trauma and whether it was sutured or not.

**Results:**

The proportion of women with an intact perineum at delivery was 9.6% (125/1,302) in nulliparae, and 31.2% (453/1,452) in multiparae, with a higher incidence in the community (freestanding midwifery-led units and home settings). Multivariable analysis showed multiparity (OR 0.52; 95% CI: 0.30–0.90) was associated with reduced odds of obstetric anal sphincter injuries (OASIS), whilst forceps (OR 4.43; 95% CI: 2.02–9.71), longer duration of second stage of labour (OR 1.49; 95% CI: 1.13–1.98), and heavier birthweight (OR 1.001; 95% CI: 1.001–1.001), were associated with increased odds. Adjusted ORs for spontaneous perineal truama were: multiparity (OR 0.42; 95% CI: 0.32–0.56); hospital delivery (OR 1.48; 95% CI: 1.01–2.17); forceps delivery (OR 2.61; 95% CI: 1.22–5.56); longer duration of second stage labour (OR 1.45; 95% CI: 1.28–1.63); and heavier birthweight (OR 1.001; 95% CI: 1.000–1.001).

**Conclusions:**

This large prospective study found no evidence for an association between many factors related to midwifery practice such as use of a birthing pool, digital perineal stretching in the second stage, hands off delivery technique, or maternal birth position with incidence of OASIS or spontaneous perineal trauma. We also found a low overall incidence of OASIS, and fewer second degree tears were sutured in the community than in the hospital settings. This study confirms previous findings of overall high incidence of perineal trauma following vaginal delivery, and a strong association between forceps delivery and perineal trauma.

## Background

About 85% of women in the UK sustain some degree of perineal trauma during childbirth [1]. Clinical diagnosis of obstetric anal sphincter injury (OASIS) comprising a third or fourth degree perineal tear occurs in about 3% of women after having their first baby, and 0.8% of women who have previously had at least one baby [2]. However, results from a systematic review indicate that the true incidence may be as high as 11% [3]. The incidence of perineal trauma varies markedly between studies with occurrence tending to be higher in hospital settings compared with community settings [4]. There is some evidence from one large UK single-centre study that the incidence has increased in recent years [5], and in Norway from 1% in late 1960s to 4.3% in 2004 [6], and Sweden from 1.7% in 1990 to 4.2% in 2004 [7]. Whether these changes are due to greater awareness and improved identification or due to an actual rise in incidence is uncertain. Interestingly, a significant decline from 4.03% in 2002 to 1.17% in 2007 in the proportion of women with anal sphincter tears was found in Norway and attributed to an intervention program involving slowing the delivery of the infant’s head and instructing the mother not to push during delivery of the head [6,8].

OASIS is associated with significant short and long term maternal morbidity. Anal incontinence is reported by 4.3% (95% confidence interval (CI): 3.5 to 5.9) of women aged 15 to 60 years [9], however, it is acknowledged that it goes unrecognised and is under-reported. Bowel symptoms in women with OASIS vary from 7.6% to 61% depending on the severity of symptoms, parity and type of injury [10-14]. A systematic review has estimated the prevalence of any post-partum urinary incontinence with vaginal delivery as 31% (95% CI: 30 to 33%), and weekly or daily incontinence as 12% (95% CI: 11 to 13%) and 3% (95% CI: 3 to 4%), respectively [15]. Sexual dysfunction [16-18], and post-partum perineal pain may also occur [9,13,19]. A large prospective survey of Swedish postpartum women reported that 8% (167/2,154) of women had not had sexual intercourse within six months after childbirth; of those with an anal sphincter injury the proportion was higher at 13.6% [20].

Factors consistently shown to be associated with perineal tears involving the anal sphincter are instrumental delivery, [5,14,21-24] with forceps associated with a higher risk than ventouse, [5,14,21,22,25-28] longer duration of second stage of labour, [7,21,25,26,28-30] nulliparity, [5,7,14,21,25,27-29] large for gestational age or birthweight [5,7,14,21,22,24,25,27],[29,31] and occipito posterior (OP) position [14,27,28].

Episiotomy as a risk or protective factor for OASIS is controversial: some studies report a reduced risk with a mediolateral incision, [5,21,22] and others are either inconclusive, [7,24,26] or report increased risk [31]. However, randomised controlled trials (RCTs) have failed to demonstrate a significant reduction in OASIS in women who received an episiotomy compared with women who did not [32,33]. The role ethnicity plays as a risk factor for OASIS is also unclear [34-36].

Few multivariable analyses are available reporting on the impact of maternal birth or pushing position, type of pushing, digital perineal stretching during second stage, ‘hands off’ delivery, or care setting, as risk factors for perineal injury [4,7,30,37]. Many of these observational studies are retrospective, and data from RCTs have failed to corroborate the findings and are limited by small sample sizes. Another limitation of studies reporting on perineal trauma is that they have been largely conducted on women in the hospital setting, and it is important to also evaluate the incidence and pattern of perineal trauma in all settings where women plan to give birth.

The aim of this large prospective study was to estimate the range of perineal trauma sustained by women with a planned singleton vaginal delivery in community and hospital settings. Additionally, to estimate the impact of a comprehensive set of potential risk factors for any spontaneous perineal trauma encompassing the full range of anterior and posterior tears, and for OASIS defined as any third or fourth degree tear.

## Methods

We conducted a prospective observational study in one NHS Trust in the South-East of England. Care settings comprised the hospital, three freestanding midwifery-led units (FMUs) and women’s homes. All women in labour expected to have a singleton vaginal delivery were recruited from May to September 2006. A sample size of about 3,000 women was anticipated based on a rate of about 7,000 births per year at that time.

Data were recorded by the midwife caring for the woman in labour using a predesigned standardised data collection sheet. After an initial pilot phase, data collection was coordinated by three link midwives.

Data were collected on maternal and obstetric characteristics: age, parity, analgesia used, length of active and passive second stage of labour (minutes), type of pushing during the second stage, maternal position(s) adopted when pushing, pushing instructions, whether digital stretching of the perineum occurred before crowning (finger inserted into vagina, with pressure downwards on the perineum to encourage maternal pushing effort), maternal position at delivery, type of delivery, delivery technique used for spontaneous birth (whether the practitioner’s hands were on or off the perineum and/or the baby’s head), whether episiotomy was performed, and whether or not shoulder dystocia occurred. Infant related characteristics recorded were position of the baby at delivery and birth weight (grams). Data on presence or absence of previous perineal trauma, and previous perineal repair were obtained by maternal self report.

Main outcome measures were the type of perineal trauma categorised as: no trauma (defined as intact perineum); labial tear to one or both labia; other anterior tears such as clitoral or urethral tears; and vaginal wall defined as posterior and/or lateral vaginal wall involvement but perineal skin intact; first degree tear involving vaginal mucosa and perineal skin; second degree tear involving the perineal skin, superficial and/or deeper perineal muscles; third degree tear to the anal sphincter affecting less than 50% of the external anal sphincter fibres (3a), more than 50% of the external anal sphincter fibres (3b) or external and internal anal sphincter rupture (3c); fourth degree tear involving complete anal sphincter rupture that extends into the anal epithelium [1]. Additional outcomes were whether or not the perineum was sutured, and whether women were transferred from their planned place of delivery (home or FMU) to the hospital, and the reason for transfer.

Data were double-entered into a database and analysed using SPSS version 17. Perineal trauma is summarised by parity and care setting using descriptive statistics. Care settings were categorized as hospital, FMU or home according to where the woman planned to deliver at labour onset (regardless of whether the woman was transferred for maternal/neonatal reasons). Regression analyses were conducted to investigate risk factors for perineal trauma using univariable and multivariable logistic regression. To improve the power of the regression analyses, we created one community setting by combining FMU and home because in the UK they provide a very similar model of care and are located away from the hospital. In addition, with the exception of age, women in the two settings were comparable with similar proportions in each category for each risk factor. This was judged based on observing over-lapping 95% CIs. Labour length for active and passive second stage were combined, maternal birth position was categorised as: upright if semi-recumbent/sitting, all fours/kneeling forward, birth stool, kneeling/standing or squatting, or lying down if left or right lateral, lithotomy or supine. Delivery technique was categorised as ‘off’ both head and perineum, or ‘on’ if hands were placed either on the baby’s head or perineum or both. Pushing instructions were categorised as directed if pushing was directed for all or some of the time, or undirected if no directions were given. We defined OASIS as any third- or fourth-degree tear with or without an episiotomy, and spontaneous tear as any anterior or posterior tear in the absence of an episiotomy.

The relationship between potential risk factors and incidence of OASIS and spontaneous perineal trauma was investigated using logistic regression. Factors were selected for addition to the regression model if it was clinically plausible that the factor may influence perineal outcome, in addition to those that have been empirically suggested as risk factors for perineal outcome if sufficient data were available for the variable. We required a base of 100 participants with a specific outcome plus 10 positives and 10 negatives for each variable added to the model [38]. Participants with missing data were excluded from the analysis. We planned to add pushing position as a covariate to the model, however many women adopted more than one position for pushing precluding clear categorisation for analysis. Ethical approval was obtained from the ORH and the School of Health and Social Care Research Ethics Committee, Oxford Brookes University. We followed the advice given by the research ethics committee at the time that consent from women was not required.

## Results

**Figure 1:**
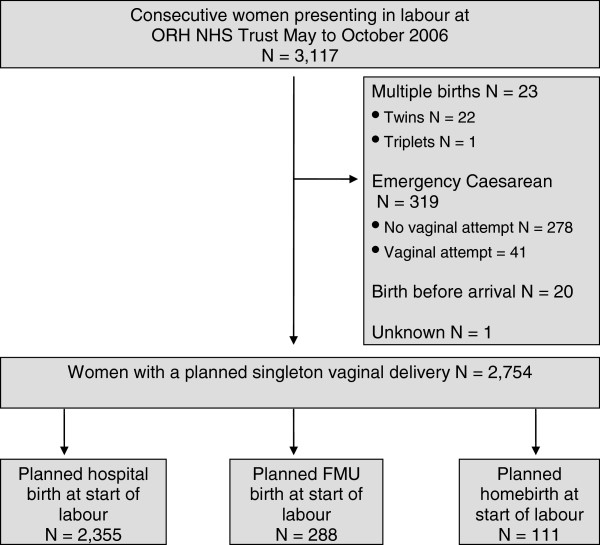
Flow diagram of participants.

Figure 1 shows the recruitment of participants to the study in the sample. We analysed data for 2,754 women with complete data for perineal outcomes; the proportion of missing data ranged from 0.3 to 7.4% with no evidence of a difference between care settings.

The majority of these women planned to deliver in the hospital (85.5%), 10.5% in an FMU and 4% at home. Among the 2,754 women, 47.3% were nulliparae, and 52.7% multiparae, with a mean age of 29.1 and 31.7 years, respectively.

### Prevalence of tears

The overall proportion of women with an intact perineum at delivery was just over three-fold higher in multiparous women, 31.2% (453/1,452) compared with nulliparae, 9.6% (125/1,302). Table 1 shows the prevalence of perineal tears by planned place of birth and parity. OASIS occurred in 6.6% (86/1,302) of nulliparae, and 2.7% (33/1,452) of multiparae overall, and occurred mainly in the hospital. The majority of OASIS cases were third-degree tears.

**Table 1:** Prevalence of perineal trauma in women with a singleton vaginal birth

	**Hospital**			**FMU**			**Homebirth**		
	**Nullip = 1,151**			**Nullip = 109**			**Nullip = 42**		
	**Multip = 1,204**			**Multip = 179**			**Multip = 69**		
	**Number with outcome**			**Number with outcome**			**Number with outcome**		
	**n**	**%**	**95% CI**	**n**	**%**	**95% CI**	**n**	**%**	**95% CI**
**Intact perineum**									
Nullip	100	8.6	7.1, 10.3	19	17.4	10.3, 24.6	6	14.3	3.6, 25.0
Multip	343	28.5	26.0, 31.0	71	39.7	32.5, 46.9	39	56.5	44.7, 68.3
**Labial tear**									
Nullip	219	19.0	16.8, 21.3	27	24.8	16.6, 33.0	12	28.6	14.7, 42.4
Multip	148	12.3	10.4, 14.2	30	16.8	11.3, 22.2	5	7.2	1.1, 13.4
**Tear of vaginal wall only**									
Nullip	62	5.4	4.1, 6.7	8	7.3	2.4, 12.3	2	4.8	0.0, 11.3
Multip*	48	4.0	2.9, 5.1	11	6.2	2.7, 9.7	1	1.5	0.0, 4.3
**First degree**									
Nullip	63	5.5	4.2, 6.8	18	16.5	9.5, 23.6	7	16.7	5.3, 28.1
Multip	147	12.2	10.4, 14.1	33	18.4	12.7, 24.0	11	15.9	7.2, 24.7
**Second degree**									
Nullip	404	35.1	32.3, 37.9	38	34.8	25.9, 43.9	12	29.0	14.7, 42.4
Multip	461	38.3	35.5, 41.0	44	24.6	18.3, 31.0	14	20.3	10.7, 29.9
**Third degree (a, b, c)**									
Nullip	69	6.0	4.7, 7.5	2	1.8	0, 4.4	3	7.1	0, 15.0
Multip	29	2.4	1.6, 3.4	1	0.6	0, 1.7	0	0	na
**Fourth degree**									
Nullip	4	0.3	0.0, 0.7	0	0	na	0	0	Na
Multip	2	0.2	0.0, 0.4	0	0	na	0	0	na
**Episiotomy**									
Nullip	364	31.6	30.0, 34.3	7	6.4	1.7, 11.5	1	2.4	0.0, 7.1
Multip	84	7.0	5.5, 8.4	0	0	na	0	0	na
**Episiotomy plus 3**^**rd**^**or 4**^**th**^**degree tear**									
Nullip	34	0.4	0.1, 0.9	0	0	na	0	0	na
Multip	5	0.0	2.0, 4.1	0	0	na	0	0	na
**Extensive tear (any 3**^**rd**^**or 4**^**th**^**degree tear with/without episiotomy)**									
Nullip	81	7.0	5.6, 8.5	2	1.8	0, 4.4	3	7.1	0, 15.0
Multip	32	2.7	1.7, 3.6	1	0.6	0, 1.7	0	0	na

Footnotes: * n = 1,203 patients analysed for hospital, and n = 178 for FMU.

Labial tears occurred more frequently in nulliparae compared with multiparae, with no evidence of a difference between care settings (Table 1). A tear involving the anterior vaginal wall occurred less frequently than labial tears: 5.5% (95% CI: 4.3 to 6.8%) nulliparae and 4.1% (95% CI: 3.1 to 5.2%) multiparae. There was no evidence of a difference between care settings (Table 1).

Table 2 shows the unadjusted and adjusted ORs for OASIS. FMU and home were pooled to create one community setting. With the exception of age, women in the two settings were comparable with similar proportions in each category for each risk factor. Women whose planned place of birth was home were slightly older (33.6 years 95% CI: 32.6, 34.7) than women whose planned place of birth was an FMU (31.8 years 95% CI: 31.2, 32.4). Risk factors shown to increase the risk of OASIS significanty in the unadjusted logistic regression analyses were nulliparity, planned hospital birth, epidural, use of ventouse, use of forceps, directed pushing, longer duration of second stage of labour, episiotomy, shoulder dystocia and birthweight. Whereas upright delivery position, hands off perineum and off head and occipito-anterior (OA) position were associated with significantly reduced risk of OASIS (Table 2). Maternal age, use of birthing pool, and digital perineal stretching before crowning was not associated with OASIS.

**Table 2:** Unadjusted and adjusted ORs for association of characteristics and OASIS in women with a singleton vaginal delivery

**Characteristics**	**Unadjusted**	**Adjusted OR**
	**OR (95% CI)**	**(95% CI)**
Nulliparity	1.0	1.0
Multiparity	0.33 (0.22, 0.50)*	0.52 (0.30, 0.90)*
Community (FMU or home)	1.0	1.0
Hospital	3.30 (1.44, 7.56)*	NE
Age group (years)^1^		
<21	1.0	
21–30	1.14 (0.44, 2.92)	NE
31–40	1.55 (0.61, 3.90)	NE
>40	1.11 (0.26, 4.76)	NE
Birthing pool^2^	1.21 (0.67, 2.18)	1.28 (0.50, 2.11)
Epidural^3^	1.78 (1.22, 2.59)*	0.56 (0.33, 0.93)*
Spontaneous (non-operative) delivery	1.0	1.0
Ventouse	2.71 (1.59, 4.61)*	2.03 (0.96, 4.30)
Forceps	6.56 (4.28, 10.1)*	4.43 (2.02, 9.71)*
Directed pushing^4^	2.66 (1.68, 4.23)*	1.15 (0.62, 2.14)
Delivery position lying down	1.0	1.0
Delivery position upright	0.47 (0.32, 0.68)*	1.17 (0.68, 2.02)
Perineum touched^5^	0.86 (0.52, 1.40)	0.91 (0.53, 1.56)
Delivery technique hands on either or both perineum and head	1.0	1.0
Delivery technique Off/off	0.48 (0.27, 0.86)*	0.77 (0.39, 1.52)
Occipito posterior or Occipito transverse	1.0	1.0
Occipito anterior	0.41 (0.22, 0.77)*	0.69 (0.33, 1.42)
Duration 2^nd^ stage (log mins)	2.10 (1.74, 2.54)*	1.49 (1.13, 1.98)*
Episiotomy^6^	2.33 (1.57, 3.46)*	0.64 (0.36, 1.15)
Shoulder dystocia^7^	7.35 (3.40, 16.0)*	NE
Birth weight (g)	1.001 (1.001, 1.001)*	1.001 (1.001, 1.001)*

Reference category: * = statistically significant (p < 0.05); 1 = age group 20 or below; 2 = birthing pool not used; 3 = no epidural used; 4 = undirected pushing; 5 = Perineum not touched; 6 = no episiotomy; 7 = no shoulder dystocia; NE = not estimated as < 10 participants with or without characteristic. Maternal birth position was categorised as: upright if semi-recumbent/sitting, all fours/kneeling forward, birth stool, kneeling/standing or squatting, or lying down if left or right lateral, lithotomy or supine. For spontaneous birth, delivery technique was categorised as ‘off’ both head and perineum, or ‘on’ if hands were placed either on the baby’s head or perineum or both. Pushing was categorised as directed if pushing was directed for all or some of the time or undirected if no directions were given.

After adjustment for all other factors with sufficient data shown in Table 2, multiparity significantly reduced the odds of OASIS, whilst use of forceps, longer duration of second stage of labour, and heavier birthweight, were associated with significantly increased odds of OASIS (Table 2). We were unable to add setting to the regression model as there were fewer than 10 events so this assessment is based on hospital deliveries. Although the initial analysis showed that epidural analgesia was associated with a significant increase in the unadjusted odds of OASIS, after controlling for the effects of other risk factors, epidural analgesia was found to be associated with reduced odds of OASIS. This is likely due to the use of epidural analgesia being associated with other risk factors, such as instrumental delivery and longer duration of second stage labour.

Table 3 shows unadjusted and adjusted ORs for spontaneous perineal trauma. Most of the risk factors were significant predictors for spontaneous perineal trauma in the univariable analyses, with the exception of parity, care setting, maternal age and shoulder dystocia (Table 3). Multivariable adjustment was conducted using all factors shown in Table 3 with the exception of maternal age group (excluded due to fewer than 10 women in some age groups with spontaneous perineal trauma). After adjusting for other factors, multiparity significantly reduced the odds of spontaneous perineal trauma, whilst hospital care setting, use of ventouse, forceps delivery, longer duration of second stage of labour, and heavier birthweight significantly increased the odds.

**Table 3:** Unadjusted and adjusted ORs for association of characteristics and spontaneous perineal trauma in women with a singleton vaginal delivery

**Characteristics**	**Unadjusted**	**Adjusted**
	**OR (95% CI)**	**OR (95% CI)**
Nulliparity	1.0	1.0
Multiparity	1.05 (0.90, 1.23)	0.42 (0.32, 0.56)*
Community (FMU or home)	1.0	1.0
Hospital	0.92 (0.74, 1.15)	1.48 (1.01, 2.17)*
Age group (years)^1^		
< 21	1.0	1.0
21–30	1.10 (0.78, 1.56)	NE
31–40	1.34 (0.81, 1.61)	NE
> 40	1.03 (0.59, 1.79)	NE
Birthing pool^2^	1.39 (1.05, 1.86)*	1.10 (0.72, 1.66)
Epidural^3^	0.69 (0.58, 0.81)*	0.90 (0.67, 1.20)
Spontaneous (non-operative) delivery	1.0	1.0
Ventouse	0.40 (0.31, 0.52)*	1.86 (1.00, 3.48)
Forceps	0.18 (0.13, 0.24)*	2.61 (1.22, 5.56)*
Directed pushing^4^	0.79 (0.67, 0.93)*	1.12 (0.88, 1.44)
Delivery position lying down	1.0	1.0
Delivery position upright	1.85 (1.56, 2.19)*	1.20 (0.93, 1.56)
Perineum touched^5^	0.91 (0.75, 1.11)*	1.09 (0.81, 1.47)
Delivery technique hands on either or both perineum and head	1.0	1.0
Delivery technique off/off	1.31 (1.08, 1.60)*	0.78 (0.61, 1.00)
Occipito posterior or Occipito transverse	1.0	1.0
Occipital anterior	2.03 (1.42, 2.91)*	1.28 (0.73, 2.24)
Duration 2^nd^ stage (log mins)	0.96 (0.90, 1.02)*	1.45 (1.28, 1.63)*
Episiotomy^6^	0.03 (0.02, 0.04) *	0.01 (0.00, 0.01) *
Dystocia^7^	0.74 (0.39, 1.41)	0.28 (0.09, 0.93) *
Birth weight (grams)	1.00 (1.00, 1.00)*	1.001 (1.000, 1.001)*

Reference category: * = statistically significant (p < 0.05); 1 = age <21 years; 2 = birthing pool not used; 3 = epidural not used; 4 = undirected pushing; 5 = perineum not touched; 6 = no episiotomy; 7 = no shoulder dystocia; NE = not estimated.

Although the multivariate analysis showed that shoulder dystocia and episiotomy were associated with a decrease in the adjusted odds of spontaneous perineal trauma (Table 3), they were associated with other risk factors, such as longer duration of second stage labour, heavier birthweight and instrumental delivery.

Not all perineal tears were sutured. For FMU, 3/46 (6.5%) nulliparae, and 3/53 (5.6%) multiparae with spontaneous perineal trauma were sutured, and none of the women who planned to have their baby at home. In hospital nearly all of the second degree tears were sutured. For nulliparae, 35.2% (31/88) first degree tears, 92.1% (418/454) second degree tears and 99.5% (370/372) episiotomies were sutured. For multiparae, 40/191 (21%) first degree tears, 462/519 (89.1%) second degree tears, 83/84 (99%) episiotomies were sutured.

Overall there were fifteen transfers to hospital, six from home and nine from an FMU. One postpartum transfer from home was for suturing of a third degree tear, four intrapartum transfers were for slow progress during labour, and one for a retained placenta and the other postpartum transfer was for neonatal concerns. Transfers from the FMU included one woman who required suturing of a complex vaginal wall tear, seven for slow progress during labour and one for a retained placenta.

## Discussion

In this large prospective study 9.6% nulliparae, and 31.2% multiparae had an intact perineum following a singleton vaginal delivery. The proportions were higher in the community (FMU and homebirth) compared with the hospital. The pattern of tears differed between settings with more first degree tears with planned community birth, compared with planned hospital birth. Reassuringly, few women sustained OASIS. The overall rate of 6.6% of nulliparae, and 2.3% of multiparae falls within estimates for anal sphincter trauma in the UK [1,3,39]. OASIS was highest among women with a planned hospital delivery; a setting that is associated with more intrapartum interventions such as operative vaginal delivery which is associated with a greater risk of perineal trauma [40].

Not all perineal tears were sutured. This reflects a trend for UK midwives not to suture some first and second degree tears. In a recent survey of UK midwives 58% reported that they did not repair second degree tears [41]. This may reflect a lack of confidence to perform a repair as has been reported in two surveys [41,42], however, a recent Cochrane review found no evidence of a difference for clinical outcomes between sutured versus non-sutured first or second degree tears [43]. There are little short- or long-term follow up data on the effects of un-sutured injury on pelvic floor function.

Multiparity was associated with a halving in the risk of OASIS, whereas forceps delivery was associated with a three and a half-fold increase in risk compared with spontaneous delivery. Longer duration of second stage of labour was associated with a 40% increase in odds of OASIS for each minute (log) increase in second stage of labour. Additionally, each 100 gram increase in birthweight was associated with a 10% increase in odds of OASIS.

Multiparity was also associated with halving the risk of spontaneous perineal trauma. Episiotomy was not associated with anterior perineal trauma, only with extensions of the episiotomy. Birthweight and duration of second stage of labour were associated with similar magnitudes of risk as for OASIS, as was forceps delivery. Hospital planned place of birth was associated with an increase (48%) in spontaneous perineal trauma compared with planned community birth, even after controlling for use of forceps or ventouse and epidural. A retrospective study also found that the risk of OASIS was significantly lower in women with a planned home birth compared with planned hospital birth: RR 0.2 (95% CI: 0.0, to 0.7) [4]. Birth environment has previously been shown to influence intrapartum interventions and outcomes including perineal trauma [44-46]. Perhaps the model of midwifery led care provided in midwifery led units, which has also been found to reduce perineal trauma [47,48], contributed to the higher rate of intact perinea that we found for nulliparae who planned to deliver in the community. However, the different case mix for the community and hospital settings may have been a contributing factor to any observed differences as the adjusted analyses would not completely account for differences in risk profiles between settings such as maternal comorbidities.

We found no evidence that episiotomy was associated with either an increased or decreased risk for OASIS. All episiotomies involved a medio-lateral incision, which is recommended practice in the UK [49]. The impact of episiotomy on OASIS is not conclusive, with many of the existing studies which reported a protective effect being of a retrospective design, thus at risk of bias due to non-standardised and incomplete data collection methods.

A large retrospective study reported a positive association between shoulder dystocia and OASIS (OR 1.8; 95% CI: 1.2, 2.9) [5]. We were unable to examine this relationship in the multivariable analysis due to insufficient women with both shoulder dystocia and OASIS. However, we found no evidence of an association between shoulder dystocia and spontaneous perineal trauma.

Anecdotal evidence has suggested that midwifery practices such as digital stimulation of the perineum before crowning or adopting hands-off during delivery may predispose women to perineal trauma. We found no evidence that these factors were associated with an increased risk of either OASIS or spontaneous tears. We also found no evidence for an association between upright and lying down birth positions for delivery, directed pushing or using a birthing pool. This is in contrast with one study that collected data retrospectively from a hospital database and reported that squatting position was independently associated with twice the risk of OASIS compared with a sitting position for delivery in women who had a non-instrumental vaginal delivery [7]. However, a Cochrane systematic review evaluating the effect of different pushing positions found no increased risk of extensive tears with squatting positions, although conclusions were limited by too few studies and participants [50]. Two further RCTs reported that perineal outcomes did not differ significantly between a kneeling or sitting upright delivery position [51], or a birthing seat and any other delivery position [52].

We found no evidence of an association between directed pushing and OASIS or any other spontaneous tear once other factors were adjusted for. This is in contrast with one study concluding directed or Valsalva pushing was associated with an increased risk of a tear requiring a suture 1.65 (95 CI: 1.05, 2.59) [53]. However, a systematic review of trials that compared directed versus undirected pushing, found no significant association between type of pushing and perineal repair, but acknowledge that the data are inconclusive [54].

A recent Cochrane systematic review of perineal procedures during labour for reducing perineal trauma found that the application of warm compresses to the perineum halved the risk of OASIS (RR 0.48, 95% CI 0.28, 0.84) based on two RCTs. Perineal massage also halved the risk of OASIS compared with no massage (RR 0.52, 95% CI 0.29, 0.94) based on two studies [55]. However we did not collect data on the use of warm compresses as during the time period of the data collection for the study, their use during the second stage of labour was not practised by midwives who were working in any of the care settings within the NHS Hospital Trust study centre. This is a factor for which data collection should be considered in future studies.

Our study has several strengths. Data were collected for all eligible women over a six month time period, so we are confident that the results can be generalised to a wider population. Data were collected prospectively by midwives caring for women during labour, ensuring standardised collection of all variables and minimisation of missing data. Also, data were collected for all perineal trauma, and a wide range of intrapartum potential risk factors for which few data have previously been reported. Moreover, we collected data representing the full range of care settings available to women in this study which included both hospital and community.

The study also has limitations that are important to consider when interpreting the data. Firstly, the risk profile of women may have differed between the different care settings and whilst we collected data on several potential risk factors for perineal trauma, we cannot exclude the possibility that we missed other potential confounding factors such as ethnicity and BMI. For some variables, such as duration of second stage labour, there is an inherent weakness in accuracy of the measurement. We were unable to evaluate some factors previously suggested as important predictors of perineal outcomes, such as having had a perineal repair for a previous birth. We found that few women reported this outcome, which is likely to be an underestimate reflecting a lack of recall rather than a true low incidence of repair, since it relied on women’s self-report. Another limitation, not unique to this study, relates to the accuracy of identification and classification of perineal trauma. It is recognised that identification based on physical examination will miss some cases of OASIS, particularly those that are difficult to see [56].

## Conclusions

This large prospective study found no evidence for an association between use of a birthing pool, maternal position for delivery and digital perineal stretching during the second stage of labour with OASIS or any other spontaneous perineal trauma. We also found a low overall incidence of OASIS and fewer second degree tears were sutured in the community compared with hospital settings. This study confirms previous findings of overall high incidence of perineal trauma following vaginal delivery and a strong association between forceps delivery and perineal trauma.

## Competing interests

The authors declare that they have no competing interests.

## Authors’ contributions

EEB designed the project, managed data collection and contributed to the analysis. LS analysed the data. CS contributed to the data analysis. All authors were actively involved in interpretation of the data and writing the manuscript. All authors read and approved the final manuscript.

## Pre-publication history

The pre-publication history for this paper can be accessed here:

http://www.biomedcentral.com/1471-2393/13/59/prepub
